# “I Don’t Want My Child to Be a Guinea Pig”: Reasons for Non-Participation in a Parental Tobacco Cessation Trial in the Pediatric Emergency Department Setting

**DOI:** 10.3390/toxics11080655

**Published:** 2023-07-28

**Authors:** Sinem Toraman Turk, Ashley L. Merianos, Lara Stone, David Schnadower, Kamali Bouvay, E. Melinda Mahabee-Gittens

**Affiliations:** 1 Department of Health Policy and Management, Global Health Leadership Initiative, Yale University, 60 College Street, New Haven, CT 06520, USA; 2 School of Human Services, University of Cincinnati, Cincinnati, OH 45221, USA; 3 Division of Emergency Medicine, Cincinnati Children’s Hospital Medical Center, 3333 Burnet Avenue, MLC 2008, Cincinnati, OH 45229, USA; lara.stone@cchmc.org (L.S.); melinda.mahabee-gittens@cchmc.org (E.M.M.-G.); 4 College of Medicine, University of Cincinnati, Cincinnati, OH 45267, USA

**Keywords:** emergency department, tobacco, parents, children, randomized control trial, consent

## Abstract

(1) Background: Pediatric emergency department (PED) settings are opportune venues in which to recruit parental smokers into tobacco cessation interventions. However, the barriers associated with parents’ participation in PED-based cessation trials are unknown. The objective was to explore parents’ reasons for non-participation in a PED-based tobacco cessation trial. (2) Methods: We employed the framework method and conducted a qualitative data analysis of parental smokers who were eligible to participate in a PED-based tobacco cessation trial and did not choose to participate (n = 371). (3) Results: Two main themes emerged about reasons for non-participation: (a) Not interested in participating in a research study, and (b) concerns specific to the study. Parents had various reasons for not participating in the cessation trial including not being interested in quitting, parents’ health and well-being, parents’ beliefs about research, and time required for the study and follow-up visits. (4) Conclusion: General disinterest and specific study-related concerns were touted as reasons for non-participation in a PED-based tobacco cessation trial. Given the potential reductions in tobacco-related morbidity to both parents and children that tobacco control interventions can facilitate, future tobacco interventions should consider alterations in study design and recruitment strategies to encourage all eligible parental smokers to participate.

## 1. Introduction

Although there is no one best approach in which to conduct intervention research, traditional randomized controlled trials (RCTs) are considered the gold standard [1]. This is because RCTs allow investigators to examine causal relationships between interventions and outcomes by eliminating much of the bias inherent in other study designs [1,2]. However, recruiting and enrolling sufficient numbers of eligible participants into RCTs can be difficult. Specifically, it can be difficult to recruit and enroll marginalized populations who may have concerns about research [3]. Further, in busy clinical venues such as Emergency Departments (EDs), due to the fast-paced nature of the visits and issues surrounding the patient’s illness and clinical flow issues, recruitment can be challenging [4,5]. Prior research indicates that it can be difficult to obtain parental consent for their child’s participation in RCTs in healthcare settings due to parents’ concerns about their child’s illness, concerns about the care their child will receive if they participate, and the required time commitment [6,7,8,9]. These reasons may explain why RCT consent rates are 50% or lower in some pediatric settings [6,9,10]. High rates of non-participation may lead to selection and sampling biases [11].

Despite these factors, the pediatric ED (PED) and urgent care (UC) settings are regarded as potentially opportune venues in which to recruit parents into tobacco cessation interventions. This is because parents who receive government insurance, are socioeconomically disadvantaged, and are active tobacco users have high rates of utilization of emergency care settings for their children’s acute and non-acute healthcare needs [5,12,13]. Thus, the recruitment of parental smokers into tobacco cessation interventions may potentially have a high impact, resulting in large decreases in adult tobacco use and reductions in pediatric tobacco smoke exposure (TSE)-related morbidity [5,14,15,16]. Despite the growing number of studies about barriers and limitations to participating in a RCT [9,17,18,19,20,21], there is a research gap in our understanding of the barriers in parents’ willingness to participate in a RCT in the PED or UC setting in which the parent and not the pediatric patient is the targeted research participant. This information is needed to maximize costly recruitment and enrollment efforts and to obtain the targeted study sample size needed to adequately assess study outcomes in RCTs [22]. Since the pediatric emergency care setting is an important venue in which to intervene with parents about issues such as their primary tobacco use behavior that may affect both their own health and their child’s health [5], a better understanding of the perceived barriers that result in study non-participation are needed. Thus, the objective of this study was to explore parents’ reasons for not participating in a tobacco cessation RCT in the PED/UC setting.

## 2. Methods

### 2.1. Study Overview, Setting, and Participants

This prospective, two-group RCT was called Healthy Families (HF; www.clinicaltrials.gov NCT02531594 accessed on 20 May 2023) [23]. HF tested the efficacy of a screening, brief intervention, and referral to treatment (SBIRT) condition plus up to 12 weeks of free nicotine replacement therapy compared to an active control condition to help parental smokers quit smoking and reduce their children’s TSE. Parents or legal guardians accompanying a 0–17-year-old child to the PED or UC sites of a large Midwestern children’s hospital were eligible to participate if they: were ≥18 years old; were a current daily smoker of combustible tobacco products (e.g., cigarettes, cigars); lived within a 50-mile radius of the PED/UC; and had a working phone number and a permanent address. Parents were excluded if: they were exclusive electronic cigarette or smokeless tobacco users; they were taking tobacco cessation medications; their child had an illness that needed immediate care and/or clinical intervention; their child was a tobacco product or marijuana user; or if they or their child could not participate for medical or cognitive reasons. Additionally, the child had to have a potential TSE-related chief complaint (e.g., cough) for which they were seeking care in the PED/UC.

Clinical research coordinators told eligible parents about the study and obtained written informed consent from interested parents and assent from children > age 11 years old. Parents completed electronic survey assessments and children had saliva, urine, and hand wipes collected during the baseline PED/UC visit. Follow-up home visits occurred 6-weeks after the baseline PED/UC visit on all participants during which similar electronic survey assessments and child sample collection occurred. Electronic or phone survey assessments also occurred 6-months after the PED/UC visit; follow-up home visits occurred if the parent reported tobacco abstinence at 6-months. If the parent declined a home visit, a mutually agreed upon location (e.g., hospital) was offered. Parents were paid up to $145 for completion of all of the components of the study. All aspects of the HF RCT were approved by the hospital’s Institutional Review Board. Further details about HF are published elsewhere [5,23].

### 2.2. Data Collection

The data were extracted from a PED/UC research database that included information on all PED/UC patients examined and/or approached for potential participation in all PED/UC clinical studies at our main hospital. This dataset was retrieved from the HF RCT database and collected from April 2016 to May 2019. For the current study, only data pertaining to the HF RCT were analyzed. Data collected and analyzed to answer this study objective was qualitative data consisting of open-ended texts about the participation status of 1012 parents of PED/UC patients who were approached for the HF RCT. This open-ended text data was collected by the clinical research coordinators at the time of the recruitment and recorded in the project database. In total, 641 consented to participate, and therefore this study focused on the qualitative data from 371 patients whose parents were approached and were eligible to participate but who declined enrollment. Additionally, we report sociodemographic and PED/UC visit-related information on each PED/UC patient whose parent was approached and were eligible to participate, but who declined enrollment. Specifically, the child’s age, sex, race, ethnicity, insurance type, and time of visit (i.e., time of day and date, season) were assessed.

### 2.3. Data Analysis

To analyze the qualitative data about reasons for non-participation in the HF RCT, we employed the framework method [24,25] following 7-stage procedures for data analysis. First, we cleaned the qualitative data and de-identified any personal information (Stage 1). Then, we familiarized ourselves with the data by reading and reviewing the full dataset (Stage 2). Next, we imported the dataset into the MAXQDA 2022 qualitative and mixed methods analysis software [26] to code the data (Stage 3) and developed a working analytical framework after consultation and discussion about our codes and categories (Stage 4). During Stage 4, two investigators (STT & LS) performed line-by-line coding on a set of 50 records together first. Then, the two investigators independently developed initial codes on a set of 100 records per person (200 records in total) inductively. For the development of the codebook, the two investigators (STT & LS) met four times, each lasting approximately an hour. During these sessions, each coder shared emerging codes and their questions. Then, three team members (STT, LS, & EMM-G) met twice, each lasting approximately an hour, for discussions to ensure consistency and address inter-rater reliability where the third investigator (EMM-G) weighed in for finalizing the codebook. Next, the two investigators (STT & LS) coded all the records by applying the codebook. Once we completed coding the data and applying the analytical framework developed by the team (Stage 5), we then charted the data into the framework matrix (Stage 6) and interpreted the data using thematic analysis [27] (Stage 7) to examine patterns of non-participation in the HF RCT. Once we developed the themes, we shared the findings with our team and the Pediatric Emergency Medicine Disparities Working Group, which is a collaborative team of researchers, educators, and clinicians seeking to address social, racial, and economic disparities in care delivery with intentional research, academic output, and direct interventions in the emergency department and in the community. We asked reflections of the group to minimize individual bias within our team. Then, the whole team met once to incorporate the feedback from the working group and engaged in group discussion where we challenged each other’s interpretation to address team reflexivity. We computed descriptive statistics (i.e., mean, standard deviation, frequencies) to examine the sociodemographic and PED/UC visit characteristics of children who did not participate in the HF RCT (Table 1).

## 3. Results

From the qualitative data analysis, two main themes emerged about reasons for non-participation in the HF RCT: (a) not interested in participating in a research study; and (b) concerns specific to the HF RCT. Each of these themes is further described below, and Table 2 presents themes, sub-themes, and exemplar quotes from the participants.

### 3.1. Not Interested in Participating in a Research Study

This overall theme focuses on parents who were not interested in participating in a research study and expressed reasons not specific to the HF RCT participation. The results showed that participants had various reasons for not being interested in participating in a research study. Accordingly, this theme consists of six sub-themes: (a) generally not interested; (b) parents not interested in quitting; (c) parents’ health and well-being; (d) parents’ beliefs about research; (e) time spent on study; and (f) time spent on follow-up visits. Each of these sub-themes is further described below.

#### 3.1.1. Generally not Interested

Of the 371 parents who did not consent to participate, 203 (54.7%) parents expressed that they were not generally interested in participating in a research study. Parents did not provide any further explanation about their disinterest.

#### 3.1.2. Parents not Interested in Quitting

Some parents shared that they were not interested in quitting smoking. Eight parents declined to participate as they did not want to receive any counseling for various reasons including being “not ready to quit and not interested” or that they did not need counseling. For example, a father said, “I can quit smoking on my own”. A mother said, “Quitting [smoking] is not an option for me, and I am not interested”. Another mother said, “I am not interested in any program to aid in smoking cessation because I am very stressed out right now; smoking helps, and I have no intention to quit smoking”. In addition, one mother pulled study staff aside and stated that she was not interested in participating as she was uncomfortable talking about smoking in front of her children.

#### 3.1.3. Parents’ Health and Well-Being

Some parents explained their disinterest in participating in a research study was due to their health and well-being. Ten parents reported “being overwhelmed” and one parent added, “I am overwhelmed and have not slept”. Four parents shared that they were “too tired to participate in a study”. One mother said, “There’s just a lot going on for me personally”. Two parents also reported having some personal health problems and therefore, they did not wish to participate in the study. For example, one mother said, “I’m dealing with some personal health problems”.

#### 3.1.4. Parents’ Beliefs about Research

Some participants reported not believing in research. Although the total number of participants (n = 3) not believing in research was small, the results indicated that their beliefs about research was rooted in their previous experiences of participating in a research study. For example, a mother stated that she had participated in research studies in the past and they were “more of a hassle than they are worth”. All three parents expressed that they did not want to “get in trouble” and they did not “believe in research”. One mother also said that she did not want her child “to be a guinea pig”.

#### 3.1.5. Timing of the Recruitment for the Study

The results also indicated that 113 (30.5%) parents did not wish to participate due to the time that would have to be spent on the study due to recruitment as they were concerned that they would have a longer PED/UC visit or stay past the time when they were discharged. One family reported that they were “in the ED for two hours and ready to go home”. Some parents expressed that they were occupied with other things and would prefer being approached later. Some parents also expressed the timing of the study being “too much”.

#### 3.1.6. Time Spent on Follow-Up Visits

Most of the parents (76.9%) did not want to participate in the study because of the time requirements and commitment involved in follow-up visits. For example, a mother did not want to participate in the study as she did not wish to complete study procedures and added, “I would not have time to complete follow-ups”. Most expressed their concerns about the “study taking too much time” and “too many steps involved in the study procedures”. Some participants also expressed that they did not want to “obligate” themselves given their other responsibilities. For example, one mother said, “I am very busy right now and cannot commit to a long study”. Some participants further explained their reasons for not being interested in follow-up visits. One mother explained that she was “too busy” to do follow-ups because she worked at two jobs, and scheduling home visits and other components would not be easy for her. Another mother expressed that she did not “want to have to deal with scheduling the follow-up home visits”. Two fathers stated they “work[ed] so hard” and it would be too hard for them to complete the study procedures including the follow-up visits. Many parents did not want to participate in the study due to their busy work schedule or because they were not comfortable with home visits. Although clinical research coordinators offered a neutral follow-up location such as the hospital, these parents still declined to participate in the study. Some parents also reported that having limited transportation to follow-up at a “neutral” site as the reason for their decline. For example, one mother said, “I do not have a car or easy transportation to come to the hospital for the follow-ups”.

### 3.2. Concerns Specific to the HF RCT

This second overall theme focuses on parents who were concerned about specific HF RCT participation. This theme consisted of three sub-themes: (a) concerns about HF RCT study procedures; (b) concerns about child health and well-being; and (c) concerns about other parent consenting. Each of these sub-themes is further described below.

#### 3.2.1. Concerns about HF RCT Study Procedures

In total, 117 (31.5%) parents shared that they had concerns about the study which could have included concerns about study procedures (e.g., sample collection, cessation intervention, follow-up visits) and therefore, were not interested in participating. Specifically, some parents expressed their concerns about the follow-up requirements and sample collection at baseline, 6-weeks, and 6-months. Four mothers declined participation in the study due to specific concerns about sample collection. For example, one mother said, “My child does not like to be touched. Sample collection would make her upset”. Another mother also did not want samples taken from the child.

#### 3.2.2. Concerns about Child Health and Well-Being

All children had a potential TSE-related chief complaint (e.g., cough) when their parents were approached about potential participation in the HF RCT during the PED/UC visit. In total, 29 (7.8%) parents expressed their concerns about their child’s health and well-being at the time of recruitment. Many parents said that they wanted to “focus on the child’s care”. For example, one mother said, “I just want to focus on [the child] getting better”.

#### 3.2.3. Concerns about other Parent Consenting

Five parents declined participation in the study due to not having the child’s other parent available at the time of the visit. For example, a mother was interested in participating in the study but said, “I need to talk with my husband, and he is not here with us. I am happy to be approached again in the future”. Similarly, two mothers wanted the child’s father to read over the consent document before agreeing to participate in the study. They expressed their concern about samples and were worried the other parent might not agree with their decision.

## 4. Discussion

The purpose of this study was to explore parents’ reasons for not participating in a tobacco cessation RCT in the PED/UC setting. The results showed that of the 1012 potential parental smokers who brought their child to the PED/UC and were eligible to participate in a free tobacco cessation and child TSE reduction trial, 37% declined to participate and were included in this study’s qualitative analysis. This percentage of non-participation in the HF RCT is concerning because the pediatric healthcare setting is an important locus of tobacco control that if used effectively and routinely, could result in reductions in tobacco-related morbidity to both parents and children [5,28,29]. Additionally, it is important to understand how to improve recruitment and enrollment rates of tobacco cessation RCTs in pediatric settings in order to adequately assess the primary study outcomes and to leverage the reductions of bias inherent in other study designs [1,2]. Thus, it is crucial to determine the best way to engage parental smokers in tobacco cessation interventions in pediatric acute care settings.

Our qualitative analysis indicated that parents who did not participate in this free HF RCT had a lack of interest in the study, concerns about specific study components, and concerns about follow-up requirements. Most prior research that has examined reasons for non-participation in the healthcare setting have focused on reasons why parents do not consent to their child’s participation in a pediatric clinical trial versus their own consent to participate [19,20,21]. Nevertheless, this prior work is relevant as it further informs the current study’s results and provides guidance on approaches that may improve recruitment of parents into future trials in the PED/UC setting. A prior systematic review found that parents were more likely to participate if they felt like the research would potentially benefit their child, if they trusted the research being conducted, and if they had a relationship with the researcher [21]. On the other hand, similar to the concerns voiced by parents in this present study, parents were less likely to participate if they felt like the research would disrupt their daily life, if there were logistical challenges such as too many visits, if the RCT was not convenient, or if parents were concerned that the research would cause their child to be uncomfortable or burdened [21].

The present study required parents to be active participants in the RCT. In order to assess parent tobacco outcomes, parents had to agree to follow-up visits at 6-weeks and 6-months after the initial PED/UC visit. Parents were asked whether they would allow their child to have biological samples collected so that child TSE outcomes could be assessed. These procedures are similar to other parental cessation trials which involved the assessment of the effects of the intervention on both the parent’s tobacco outcomes and child’s TSE outcomes [30,31,32]. Further, the present study included incentives and convenient home visits, as these may decrease study-related burdens [17]. Other reasons not assessed in this study, but that were reported as reasons why parents consented for their child to be enrolled in other pediatric studies included: parents’ interest in being more involved in their child’s clinical treatment; parents being approached by clinical or research staff whom they trusted or had a relationship with; and parents’ belief that participation would benefit their child [20]. Therefore, future parental tobacco cessation and child TSE reduction trials should consider: (a) tailoring the recruitment strategies to have racially/ethnically diverse study staff approach the parent earlier in the visit; (b) including medical staff who can explain the study procedures to the parent and who may alleviate any research-related and clinically-related concerns that parents may have; (c) providing information about how the RCT will personally and more generally benefit parents and their children; and (d) engaging potential participants in recruitment strategies prior to launching studies to determine how to best develop trust related to all aspects of the proposed research [33].

The strengths of this study include the large sample size and the examination of reported reasons for non-study participation in a large RCT in a PED/UC. However, limitations need to be acknowledged, which includes the lack of generalizability since this study was conducted at a single children’s hospital. While we were able to examine open texts of parents who declined participation, focused interviews of parents who declined would have provided more informative data on reasons for non-participation. We did not collect reasons for participation among parents who participated in the RCT, which comparing reasons for non-participation versus participation among parents is suggested in further research to inform future recruitment efforts. Additionally, we did not have access to parents’ demographic characteristics, or their tobacco use patterns or motivation to quit, as these and other factors such as preferred incentive amounts, type of follow-up methods, and preferred intervention and study components may have been associated with study participation. Thus, future work should consider using mixed methods research (collecting, analyzing, and integrating qualitative and quantitative data) on these factors in parents who decline to participate to better understand factors influencing sample and selection bias in ED settings and/or in studies requiring parental consent and child assent.

In conclusion, this study indicates that general disinterest and specific study-related concerns were touted as reasons for non-participation in this tobacco cessation intervention. Future studies should consider study design and recruitment strategies that are recommended to improve recruitment and enrollment rates such as simple explanations about the purpose and benefits of research study by medical staff; assurance that the study procedures are non-invasive and will not affect clinical care; and the inclusion of preferred incentives, brief time requirements, short follow-up windows, and liberal opportunities for follow-up methods that include flexible, participant-preferred modalities (e.g., text messaging, email) and locations (e.g., home visits) [9,17,21].

## Figures and Tables

**Table 1:** Demographic and PED/UC visit characteristics of parents of children who did not participate in the HF RCT (N = 371).

	Did Not Participate in HF (N *=* 371)
*Child Age*	
Mean (SD)	4.9 (4.8)
*Child Sex*	
Female	180 (48.5%)
*Child Race*	
Black or African American	200 (53.9%)
White	169 (45.6%)
Other	2 (0.5%)
*Child Ethnicity*	
Non-Hispanic	362 (97.6%)
*Child Insurance Type*	
Commercial	50 (13.5%)
Government/Self-pay	321 (86.5%)
*Day of the Week Recruited*	
Weekdays	348 (93.8%)
Weekends	23 (6.2%)
Time of the Day Recruited *	
Day (8:00 am–4:59 pm)	341 (91.9%)
Evening (5:00 pm–11:59 pm)	30 (8.1%)
*Season Recruited*	
Autumn	58 (15.6%)
Winter	90 (24.3%)
Spring	114 (30.7%)
Summer	109 (29.4%)

Note: HF = Healthy Families; SD = Standard deviation. * Day and evening time did not alternate by seasons.

**Table 2:** Themes, Sub-themes, and Exemplar Quotes.

Theme	Sub-Theme	Exemplar Quote
Theme 1: Not Interested in Participating in a Research Study	Generally Not Interested	“I am just not interested”.
	Parents Not Interested in Quitting	“Quitting [smoking] is not an option for me, and I am not interested”.
	Parents’ Health and Well-Being	“I’m dealing with some personal health problems”.
	Parents’ Beliefs about Research	“I participated in research before. They are more of a hassle than they are worth”.
	Timing of the Recruitment for the Study	“We are [have been] in the ED for two hours and [are] ready to go home”.
	Time Spent on Follow-up Visits	“I do want to have to deal with scheduling the follow-up home visits”.
Theme 2: Concerns Specific to the HF RCT	Concerns about HF RCT Study Procedures	“My child does not like to be touched. Sample collection would make her upset”.
	Concerns about Child Health and Well-Being	“I just want to focus on [the child] getting better”.
	Concerns about Other Parent Consenting	“I need to talk with my husband, and he is not here with us. I am happy to be approached again in the future”.

## Data Availability

The dataset will be made available upon reasonable request for the purpose of reproducing the findings.
